# Small molecule inhibitor of OGG1 blocks oxidative DNA damage repair at telomeres and potentiates methotrexate anticancer effects

**DOI:** 10.1038/s41598-021-82917-7

**Published:** 2021-02-10

**Authors:** Juan Miguel Baquero, Carlos Benítez-Buelga, Varshni Rajagopal, Zhao Zhenjun, Raúl Torres-Ruiz, Sarah Müller, Bishoy M. F. Hanna, Olga Loseva, Olov Wallner, Maurice Michel, Sandra Rodríguez-Perales, Helge Gad, Torkild Visnes, Thomas Helleday, Javier Benítez, Ana Osorio

**Affiliations:** 1 Human Genetics Group, Human Cancer Genetics Programme, Spanish National Cancer Research Centre (CNIO), 28029 Madrid, Spain; 2 Science for Life Laboratory, Department of Oncology-Pathology, Karolinska Institutet, 17121 Solna, Sweden; 3 Molecular Cytogenetics Group, Human Cancer Genetics Programme, Spanish National Cancer Research Centre (CNIO), 28029 Madrid, Spain; 4 Department of Biomedicine, School of Medicine, Josep Carreras Leukemia Research Institute, University of Barcelona, 08036 Barcelona, Spain; 5 Department of Oncology and Metabolism, The Medical School, The University of Sheffield, Sheffield, S10 2RX UK; 6 Department of Biotechnology and Nanomedicine, SINTEF Industry, 7465 Trondheim, Norway; 7 Spanish Network on Rare Diseases (CIBERER), 28029 Madrid, Spain; 8 Human Genotyping-CEGEN Unit, Human Cancer Genetics Programme, Spanish National Cancer Research Centre (CNIO), 28029 Madrid, Spain

**Keywords:** DNA damage and repair, Telomeres, Cancer therapy

## Abstract

The most common oxidative DNA lesion is 8-oxoguanine which is mainly recognized and excised by the 8-oxoG DNA glycosylase 1 (OGG1), initiating the base excision repair (BER) pathway. Telomeres are particularly sensitive to oxidative stress (OS) which disrupts telomere homeostasis triggering genome instability. In the present study, we have investigated the effects of inactivating BER in OS conditions, by using a specific inhibitor of OGG1 (TH5487). We have found that in OS conditions, TH5487 blocks BER initiation at telomeres causing an accumulation of oxidized bases, that is correlated with telomere losses, micronuclei formation and mild proliferation defects. Moreover, the antimetabolite methotrexate synergizes with TH5487 through induction of intracellular reactive oxygen species (ROS) formation, which potentiates TH5487-mediated telomere and genome instability. Our findings demonstrate that OGG1 is required to protect telomeres from OS and present OGG1 inhibitors as a tool to induce oxidative DNA damage at telomeres, with the potential for developing new combination therapies for cancer treatment.

## Introduction

Telomeres are nucleoprotein structures that protect the ends of linear eukaryotic chromosomes. In humans, telomeric DNA is usually of 10–15 kb in length, and is composed of tandemly 5′-(TTAGGG) n-3′ hexanucleotide repeats that are coated by the telomere shelterin complex^1,2^. Functional telomeres maintain genome stability by preventing chromosomal ends from being recognized as DNA strand breaks^3^. On the other hand, dysfunctional telomeres, arising from the loss of telomeric repeats and/or sheltering protection, are recognized by many DNA damage response proteins, including the phosphorylated H2A histone family member X at serine 139 (γH2AX) or the p53-binding protein 1 (53BP1), that form telomere dysfunction-induced foci (TIF) and lead to genomic instability, cell proliferation defects or apoptosis^4,5^. Cancer cells are characterized by preserving stable telomere length, thereby conferring cell immortality^6^. In consequence, the induction of telomeric instability is considered as a potential therapeutic strategy for cancer treatment^7^.

Oxidative DNA damage is generated by reactive oxygen species (ROS) and constitutes the majority of DNA damage in human cells^8^. Several lines of evidence indicate that telomeres are particularly sensitive to oxidative stress (OS)^9,10^. Considering that guanine has the lowest redox potential among canonical nucleobases, the high incidence of these residues in the telomeric DNA sequence makes telomeres a potential hotspot for oxidative DNA damage^11^. In OS conditions, 8-oxoguanine (8-oxoG) is the most common base lesion, and can be converted into single or double-strand breaks (SSBs or DSBs), if it is not repaired correctly, or can be mutagenic by GC:TA transversions^12^. At telomeres, 8-oxoG decreases the binding of the shelterin complex^13,14^, potentially leading to telomere shortening, uncapping and finally telomere crisis^14–16^. This is a cellular state characterized by extensive genomic instability, including translocations, amplifications, and deletions related to aging-induced processes and cancer^17^.

The Base Excision Repair (BER) pathway is the main responsible for removing oxidized nucleotides from DNA, and it is active at telomeres^18,19^. BER can be initiated by eleven different DNA glycosylases that recognize and excise specific base lesions. In the case of BER initiation due to 8-oxoG excision by 8-oxoG DNA glycosylase 1 (OGG1), the resulting abasic sites are cleaved by an apurinic/apyrimidinic endonuclease (APE1), that generates a nucleotide gap containing a 3′-hydroxyl end. In short-patch BER the nucleotide gap is filled up with the correct nucleotide by polymerase β (POLB) and sealed by DNA ligase III (LIG3), in a process mediated by the scaffold protein X-ray repair cross-complementing 1 (XRCC1)^20,21^. In human cells, 8-oxoG is mainly excised by OGG1, which removes 8-oxoG opposite cytosine in double-stranded DNA. This enzyme is necessary to preserve telomere integrity, especially under OS conditions^14,16,22^.

Fouquerel et al.^16^ have recently proven that acute 8-oxoG telomeric formation in cells lacking functional OGG1 leads to telomere fragility, while a chronic 8-oxoG exposure results in telomere shortening, replication stress at telomeres, telomere losses and postmitotic defects such as micronuclei formation, anaphase bridge formation, chromosome fusions and proliferation defects^16^.

The recently developed OGG1 inhibitor TH5487 has proved to bind efficiently to the catalytic site of OGG1 blocking its enzymatic activity^23,24^. Cultured cells treated with TH5487 are more sensitive to OS conditions^23^. Surprisingly, the absolute levels of genomic 8-oxoG remain low after TH5487 treatment^25^, however its impact on specific genome locations enriched in 8-oxoG has not been studied. We hypothesize that OGG1 inhibition may recapitulate the phenotypic telomeric defects previously observed in OGG1 depleted cells^16^, representing an attractive and unexplored opportunity for compromising telomere integrity in cells exposed to high ROS levels. Furthermore, OGG1 inhibitors may potentiate the telomere instability associated with conventional chemotherapeutic agents, increasing their therapeutic effect.

In the present study, we characterize temporal-space OGG1 DNA repair activity at telomeres of cancer cells during basal and OS conditions. Secondly, we explore whether OGG1 inhibition can interfere with BER activation at telomeres and characterize the telomere and cellular defects associated with OGG1 inhibition or depletion. Finally, we perform a screening of conventional chemotherapeutic agents that might synergize with OGG1 inhibitors contributing to telomere or genome instability.

## Results

### Telomeres are a hotspot for oxidation due to cell cycle ROS production

**Figure 1:**
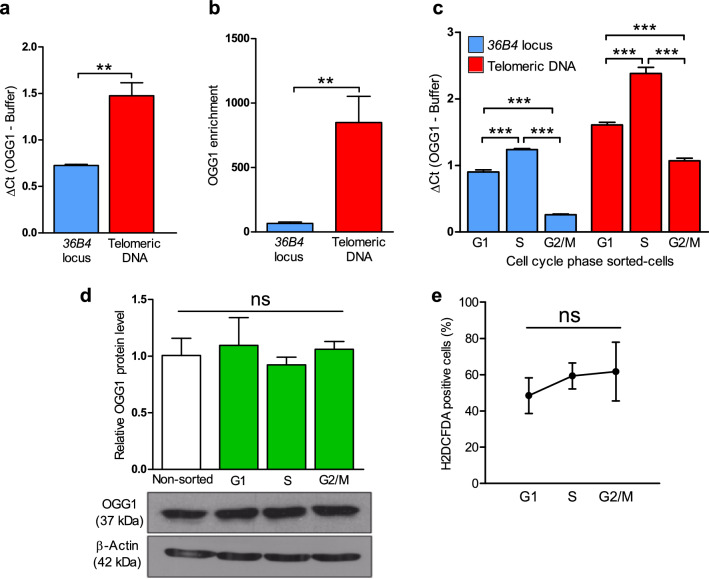
Telomeres are a hotspot for oxidation due to cell cycle ROS production. (**A**) DNA from U2OS cells was used to measure 8-oxoG levels at the *36B4* locus or telomeric DNA. Bars show the mean and the standard error of the mean (SEM) from 3 technical replicates from 6 independent experiments for each condition (Mann–Whitney test; **P < 0.01). (**B**) OGG1-GFP pulldown followed by chromatin immunoprecipitation coupled to PCR for amplification of either *36B4* locus or the telomere regions in the U2OS parental and U2OS OGG1-GFP cell lines. Data are the average of 3 technical replicates from 2 independent experiments. Statistical significance was determined using (Mann–Whitney test; **P < 0.01. (**C**) Sorted DNA from different cell cycle phases (G1, S, or G2/M) from U2OS cells was used to evaluate 8-oxoG levels at the *36B4* locus or telomeric DNA along the cell cycle. Bars show the mean and the SEM from 3 technical replicates from 6 independent experiments for each condition (two-sided T-test; *** P < 0.001). (**D**) Quantification of OGG1 protein expression level in U2OS cells along the cell cycle. Actin levels were used to normalize for protein loading. Immunoblotting was performed in triplicate. Statistical significance was determined by two-sided T-test (P > 0.05, ns: not significant). The full-length blots are presented in Supplementary Figure S2 (**E**) Percentage of cells with intracellular ROS levels above the median of the whole U2OS population is represented in different cell cycle stages (G1, S, or G2/M). Data are average ± SEM from single measures from 3 independent experiments. Significant differences were addressed by two-sided T-test (P > 0.05, ns: not significant).

We used the U2OS osteosarcoma cell line, a well-established model in telomere biology^26^, for most experiments presented in this study. By using a modified version of the procedure described by O’Callaghan et al.^27^, we measured by qPCR oxidative DNA damage at telomeric DNA or the *36B4* locus, whose amplified regions contain a similar percentage of G:C base pairs (50% telomeres vs 52% *36B4*). In basal conditions, we found that U2OS cells accumulate higher levels of oxidized bases at telomeres compared to *36B4* locus (Fig. 1A). Complementary, chromatin immunoprecipitation (ChIP) coupled to telomere PCR showed that OGG1 was significantly enriched at telomeres compared to the genomic region *36B4* in basal conditions (Fig. 1B). Then, we measured oxidized bases levels in DNA from cells sorted by cell cycle phase. In both genomic regions, the highest level of oxidative DNA damage was detected in the S phase (Fig. 1C). The highest relative levels of telomeric oxidized bases between telomeres and the *36B4* locus (ΔCt ratio telomeres/36B4) were found during the G2/M phase (Supplementary Figure S1C).

Interestingly, OGG1 protein levels remained constant throughout the cell cycle in U2OS cells (Fig. 1D). In contrast, when we measured intracellular ROS production, we observed that in the transition from G1 to S phase, endogenous ROS levels increased until reaching maximum values during G2/M (Fig. 1E), coinciding with the highest relative level of oxidized bases detected at telomeres. These results suggest that oxidative DNA damage generated by endogenous ROS might be progressively accumulated in telomeres throughout the cell cycle.

### OGG1 initiates BER at telomeres upon OS

In order to generate OS conditions, U2OS cells were exposed to H_2_O_2_ treatment (200 µM/1 h). This treatment significantly increased oxidative lesions at telomeric DNA (Supplementary Figure S3A), demonstrating its efficacy. However, we measured OGG1 protein levels (Supplementary Figure S3B) under OS conditions, and no significant differences were detected compared to untreated cells.

Confocal microscopy in U2OS cells expressing OGG1 fused to green fluorescent protein (GFP; OGG1-GFP) was used to track whether OGG1 was recruited to damaged DNA after OS treatment. First, we removed the chromatin-unbound OGG1-GFP by using a pre-extraction step before fixation. Nuclear patches were detected only in cells exposed to OS, while in non-treated cells all the OGG1-GFP signal was removed (Supplementary Figures S3C and S3D). This result reflects the OGG1 recruitment to chromatin in response to oxidative DNA damage. Interestingly, suspension of OS treatment followed by a recovery period (fresh medium/1 h), caused a reduction in the levels of oxidized bases at telomeres (Supplementary Figure S3A), together with a significant decrease in OGG1-GFP recruitment to the DNA (Supplementary Figures S3C and S3D), evidencing the repair of oxidative lesions by BER. Furthermore, we measured by immunofluorescence (IF) OGG1-GFP and XRCC1 signal intensity within Telomeric Repeat-binding Factor 2 (TRF2) foci (Supplementary Figures S3E–S3H). OS treatment significantly increases OGG1 and XRCC1 at TRF2 foci, providing evidence of BER activation at telomeres.

### Pharmacological OGG1 inhibition disrupts BER at telomeres upon OS

The novel OGG1 inhibitor TH5487 has been reported to bind the active site of OGG1, blocking any potential interaction with its natural substrate (8-oxoG) in DNA^23^. After showing that OGG1 is required to initiate BER at telomeres, we evaluated whether pharmacological inactivation of OGG1 using TH5487 may block BER at telomeres. Firstly, we confirmed that TH5487 engaged OGG1 in U2OS cells by thermal shift assay (Supplementary Figure S4A). Next, we challenged U2OS cells with TH5487 (10 µM) and measured every 24 h for 4 days the level of oxidized bases at telomeres. We found that TH5487 led to a slow and progressive accumulation of oxidized bases (Supplementary Figure S4B), likely derived from the ability of TH5487 to preclude OGG1 binding to the damaged telomeres (Supplementary Figure S4C).

Complementary, U2OS OGG1-GFP cells were used to generate a knockout (KO) for the *OGG1* gene by CRISPR/Cas9 (OGG1-KO, see material and methods). OGG1-KO efficacy was validated by fluorescence microscopy and immunoblotting (Supplementary Figures S4D and S4E). OGG1-KO U2OS cells were used to compare the effects between OGG1 depletion and inhibition with TH5487 at telomeres. Similarly to the treatment with the OGG1 inhibitor, OGG1-KO cells showed increased levels of oxidized bases at basal conditions at telomeres or *36B4* locus compared to U2OS parental cells (Supplementary Figure S4F).

**Figure 2:**
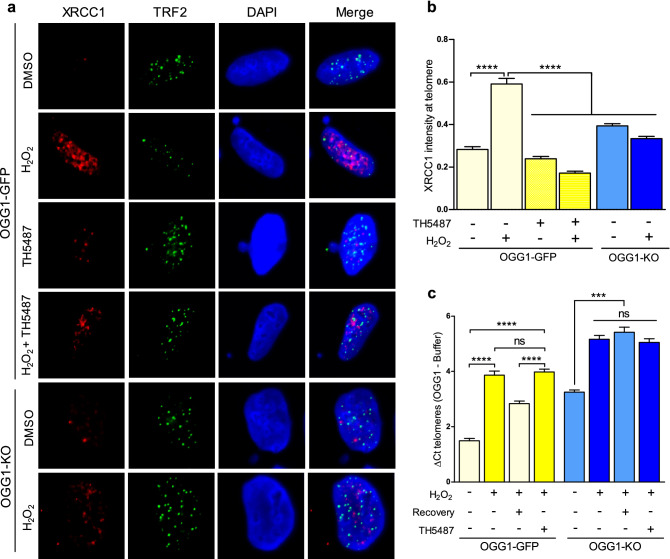
Pharmacological OGG1 inhibition disrupts BER at telomeres upon OS. (**A**) Confocal imaging of XRCC1 (red) and TRF2 (green) by IF using specific antibodies. DAPI was used to stain the cell nucleus (blue). (**B**) Quantification of XRCC1 signal intensity integrated within telomeres from more than 200 foci per condition. Data represent average with SEM from 2 independent experiments (Mann–Whitney test; **** P < 0.0001). (**C**) Relative level of oxidized bases at telomeres in OGG1 inhibited/depleted U2OS cells upon OS treatment (H_2_O_2_ 200 µM/1 h) or followed by a recovery period (fresh media/1 h). Bars show the mean and the SEM from 3 technical replicates from 6 independent experiments for each condition (two-sided T-test; *** P < 0.001, ns: not significant).

Additionally, we evaluated by IF the ability of TH5487 to inhibit BER at telomeres. Both in basal and upon OS treatment, OGG1 inhibition (TH5487) or depletion (OGG1-KO cells) resulted in a decrease of XRCC1 signal intensity at the telomere (Fig. 2A,B), which reflects BER disruption at this specific region. In order to determine the consequences of BER impairment, we measured in these cells the levels of oxidized bases at telomeric DNA by qPCR compared to the OGG1 proficient cells. We confirmed that OGG1 depletion resulted in a higher accumulation of oxidative DNA damage at telomeres, particularly exacerbated upon OS (Fig. 2C). Furthermore, a recovery period (fresh medium/1 h) after OS treatment, which is coupled to a decrease in oxidative base lesions at telomeric DNA from U2OS proficient cells, did not alleviate oxidative DNA damage accumulation for OGG1-KO cells or OGG1 proficient cells treated with TH5487. Besides, unlike in the case of OGG1-proficient cells, the treatment with TH5487 did not increase oxidized base lesions in OGG1-KO cells, ruling out the possibility for off-target effects of TH5487 at the telomeric DNA (Fig. 2C).

Finally, we measured the overlapping index of DNA damage markers γH2AX and 53BP1 with TRF2 (telomere) by IF. We established two different OS conditions in the presence or absence of TH5487. For γH2AX we exposed cells to OS (H_2_O_2_ 200 µM/1 h), while in the case of 53BP1 after an initial pulse of OS (H_2_O_2_ 200 µM/1 h), we allowed cells to recover for 16 h since unrepaired SSBs generated via BER can be converted into DSBs after DNA replication^28^. Upon oxidative conditions, we found a significant increase in the overlapping index of both γH2AX and 53BP1 foci with telomere TRF2 foci. However, no significant differences were detected regarding OGG1 inhibition/depletion in basal or under OS conditions (Supplementary Figure S5).

### Pharmacological OGG1 inhibition results in telomere losses and post-mitotic defects

**Figure 3:**
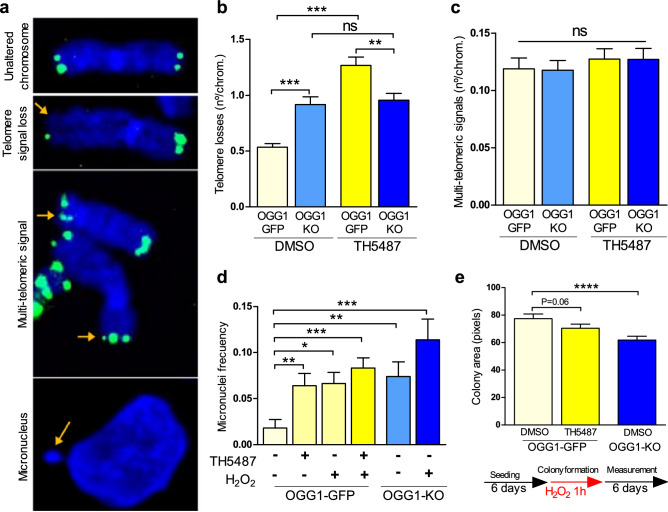
Pharmacological OGG1 inhibition results in telomere losses and post-mitotic defects. (**A**) Representative Telo-FISH images of an unaltered chromosome in metaphase stained with DAPI (in blue) with the corresponding telomeric signals (in green) at the end of each chromatid. Below, Representative Telo-FISH images of altered chromosomes showing telomere signal loss in one of the chromatids, multi-telomeric signals, and a micronucleus (orange arrows). (**B**) Quantification of telomeric signal-free ends for the indicated conditions in U2OS OGG1-GFP or OGG1-KO cells. Bars show the mean and the SEM for frequency events/metaphase (30 to 35 metaphases per condition from 2 independent experiments). Statistical significance was determined using unpaired, two-sided T-tests (**P < 0.01, ***P < 0.001 and ns: not significant). (**C**) Comparative analysis of the frequency of multi-telomeric signals for the indicated conditions in U2OS OGG1-GFP or OGG1-KO cells. Bars show the mean and the SEM for frequency events/metaphase (30 to 35 metaphases per condition from 2 independent experiments). Statistical significance was determined using unpaired, two-sided T-tests (P > 0.05, ns: not significant). (**D**) Comparative analysis of micronuclei formation frequency for U2OS OGG1-GFP incubated with DMSO or TH5487 and for OGG1-KO at basal conditions or during OS. More than 200 cells per condition were analyzed. Data is the average of 2 independent experiments. Significant differences were calculated using the Mann–Whitney test for non-parametric distributions (**P < 0.01, ***P < 0.001, **** P < 0.0001). (**E**) Up, comparative analysis for the colony area generated in each condition. Significant differences were calculated using the Mann–Whitney test for non-parametric distributions (**** P < 0.0001). Down, summary for the schedule of treatment to evaluate a specific clonogenic potential feature (area of colonies). Data are average of the mean colony area values of a single experiment.

To study the consequences of BER disruption at telomeres by OGG1 inhibition, we examined by telomere fluorescence in situ hybridization (Telo-FISH) whether TH5487 compromised telomere integrity, or led to post-mitotic abnormalities affecting cell proliferation. Analysis of metaphase chromosomes from U2OS OGG1-GFP cells revealed that 24 h of exposure to TH5487 in U2OS was enough to observe a significant increase in telomere losses (signal-free ends) compared to the control treatment with dimethyl sulfoxide (DMSO). Similarly, OGG1-KO cells presented a higher number of telomere losses than OGG1-GFP cells while TH5487 treatment did not cause additional telomere losses in OGG1-KO cells, excluding off-target effects (Fig. 3A,B). On the contrary, we did not observe significant differences in telomere fragility (multi-telomeric signal) after OGG1 depletion or inhibition at basal conditions (Fig. 3A,C). Upon OS treatment, we observed a significant increase in the frequency of chromosomes with multi-telomeric signals in both groups regardless of the OGG1 status, and no additional effect on telomere losses (Supplementary Figures S6A and S6B). These results suggest that telomere losses might be associated with OGG1 deficiency, while telomere fragility is a general phenotype occurring in OS conditions.

It has been previously reported that the accumulation of oxidative DNA damage induces replication stress at telomeres leading to post-mitotic defects, especially when OGG1 is knocked down^16^. Here, we measured micronuclei and chromosome bridge formation to evaluate whether exposure to OS in OGG1 depleted or inhibited cells have effects on genomic stability. We found that OS caused a significant formation of micronuclei in both OGG1 proficient and deficient cells (Fig. 3D). Interestingly, OGG1-KO cells or OGG1 proficient cells treated with TH5487 presented a significantly higher frequency of micronuclei regardless of OS conditions. In contrast, we were not able to find enough chromosome bridges to make a comparative analysis and, only in OGG1-KO cells, we could find them at a very low frequency (0.007).

Finally, we evaluated whether OGG1 inhibition or depletion impaired clonogenic potential in U2OS cells. No effect due to the OGG1 status was detected (Supplementary Figure S6C), reflecting that OGG1 inhibition/depletion is not enough to arrest cell proliferation in U2OS cells. Nevertheless, we found that when we inflicted OS conditions transiently during colony formation (H_2_O_2_ 200 µM/1 h 6 days after seeding), OGG1 inhibition in combination with OS lead to the formation of smaller colonies compared to OGG1 proficient U2OS cells (P = 0.06), a phenotype that was significantly more pronounced in the OGG1-KO cells (Fig. 3E). These results indicate that upon OS conditions, OGG1 inhibition/depletion may lead to proliferation defects.

### TH5487 synergizes with conventional anticancer drugs through induction of intracellular ROS, telomere DNA damage, and genome instability

We carried out a screening searching for potential synergies between OGG1 inhibitors and conventional chemotherapeutic drugs (cisplatin, 5-Fluoracil, doxorubicin, methotrexate) or other BER inhibitors (olaparib, APE1i) which might potentiate some of the phenotypes described for TH5487 alone with an impact on cell viability for different human cell lines (U2OS, BJ-TERT, NTUB1, and HCT116).

First, we tested the effect on viability of different drug combinations during 72 h in the different cell lines and calculated synergy scores (Table 1). We found that for all the drugs tested, methotrexate was the only compound that in combination with TH5487 presented a Z-score indicative of true synergy (Z-Score > 10, https://synergyfinder.fimm.fi/) for all tested cell lines (Table 1).

**Table 1:** ZIP synergy scores for conventional chemotherapeutic drugs or BER inhibitors in combination with TH5487.

Cell line	Methotrexate	Doxorubicin	5-Fluoracil	APE1i	PARPi	Cisplatin
HCT116	10.77	4.97	5.33	3.55	3.09	2.84
NTUB1	48.56	5.84	− 3.09	3.05	3.28	− 14.97
U2OS	11.36	2.891	0.5	− 2.57	1.97	− 13.28
BJ-TERT	21	5.88	–	–	− 7.46	–

**Figure 4:**
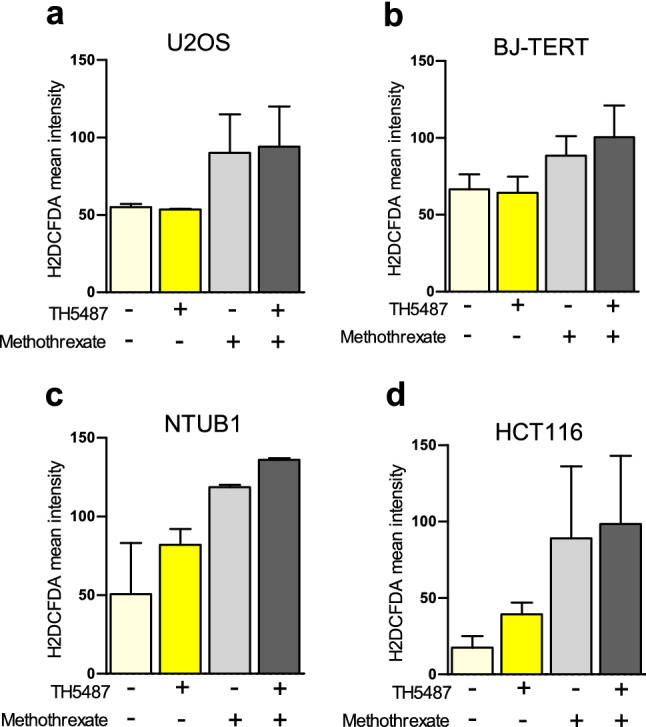
Intracellular ROS formation. (**A**) Intracellular ROS (H2DCFDA mean intensity) generated after 72 h of exposure to methotrexate (10 µM), alone or in combination with TH5487 (10 µM) in U2OS cells, (**B**) BJ-TERT, (**C**) NTUB1 or (**D**) HCT116 cells. 10 µM of TH5487 were used in combination with methotrexate for all the cell lines, except for NTUB1 where 5 µM of TH5487 were used. Data are average and SEM of single measurements from 2 independent experiments. Statistics were excluded from this analysis due to the low amount of data points to establish relevant comparisons between groups.

Next, based on the synergy maps generated with the viability data (Supplementary Figure S7), we selected sublethal doses within the synergy area for TH5487 (5–10 µM), and methotrexate (10 µM) to evaluate the effect of each drug individually or in combination, on intracellular ROS generation, micronuclei formation, and 53BP1 foci overlapping index with TRF2 (telomeres). We first measured the intracellular ROS generated after 72 h of exposure to methotrexate alone, or in combination with TH5487 in four different cell lines (Fig. 4). Intracellular ROS formation by TH5487 was cell line-specific. In contrast, methotrexate induced intracellular ROS formation was detected in all cell lines tested at different levels. In combination with TH5487, only in NTUB1 and in HCT116 cell lines an additive interaction for intracellular ROS formation was detected (Fig. 4).

**Figure 5:**
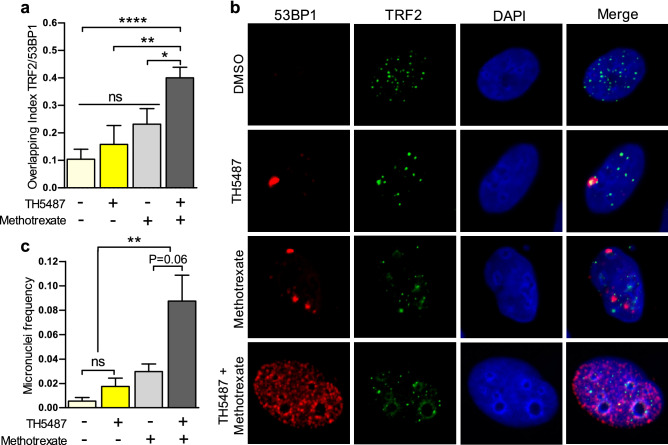
TH5487 synergizes with methotrexate through induction of telomere DNA damage, and genome instability. (**A**) Quantification of 53BP1 signal intensity integrated within telomeres from more than 200 U2OS cells per condition. Data are the average with SEM from 2 independent experiments. Significant differences were calculated using the Mann–Whitney test for non-parametric distributions (**** p < 0.0001 and ns: not significant). (**B**) Confocal imaging at single U2OS cells representative for each treatment condition and stained for 53BP1 (red) and TRF2 (green) using specific antibodies or DAPI to stain cell nucleus (blue). (**C**) Comparative analysis of micronuclei formation frequency for U2OS incubated with methotrexate (10 µM) for 72 h, alone or in combination with TH5487 (10 µM) in U2OS cells. More than 200 cells per condition were analyzed. Data is the average of 2 independent experiments. Significant differences were calculated using the Mann–Whitney test for non-parametric distributions (** p < 0.01 and ns: not significant).

Next, we tested whether methotrexate alone or in combination with TH5487 could induce 53BP1-TIF or micronuclei formation in U2OS, BJ-TERT, and NTUB1. We excluded from the analysis HCT116 due to a lack of telomeric TRF2 foci detection by IF. In U2OS, TH5487 in combination with methotrexate caused a global effect (increased 53BP1/TRF2 overlapping index and increased micronuclei formation) greater than the sum of the individual effects of each drug, suggesting that TH5487 and methotrexate are synergistic drugs for these specific phenotypes (Fig. 5).

In the case of NTUB1 cell line, methotrexate increased significantly 53BP1 and TRF2 overlapping index, and TH5487 potentiated the effect of methotrexate in an additive manner, while micronuclei formation remained unchanged with any drug combination (Supplementary Figures S8A and S8B). In contrast, neither 53BP1 foci formation at telomeres nor micronuclei formation were detected in BJ-TERT cells after exposure to TH5487 or methotrexate alone or in combination (Supplementary Figures S8C and S8D).

## Discussion

Telomeres and telomerase are key biological structures that facilitate cancer cell proliferation since cancer cells can bypass the lifespan limits of normal cells by overexpressing telomerase (Telomerase +), or by using the alternative lengthening of telomeres (ALT + cells). Hence, targeting telomeres or telomerase has been classically considered as a strategy for developing cancer therapies^7^. In the present study, we have confirmed that TH5487 blocks BER initiation at telomeres in response to OS, and we have characterized some of the TH5487 related telomeric and non-telomeric phenotypes in U2OS cells. Finally, we have tested different conventional chemotherapeutic agents in combination with TH5487, to identify potential synergistic drug combinations.

Even in basal conditions, we have found that telomeres are more prone to accumulate oxidative DNA damage than other genomic regions, especially during the S and G2/M cell cycle phases (Fig. 1A and Figure S1C). This finding could be partially explained by, not only the progressive increase in intracellular ROS during the cell cycle^29,30^, (Fig. 1D) but also by the high condensation degree of telomeric chromatin during most cell cycle phases^31,32^, since OGG1 does not operate in single-stranded or G4 contexts^16,33^. In this regard, it has been recently reported that G4 folding occurs preferentially during G1/S and S-phases in U2OS cells^34^. This correlates with the maximum peak in intracellular ROS formation and with the accumulation of oxidative DNA damage detected at both genomic and telomeric regions, which might support a cell cycle control of BER through the chromatin accessibility via G4 folding/unfolding.

We used H_2_O_2_ to mimic OS conditions at telomeres^35^ and confirmed a significant increase in oxidative base lesions in the telomeric DNA (Supplementary Figure S3D). Interestingly, OGG1 protein levels did not increase in response to OS (Supplementary Figure S3A) and also remained constant throughout the cell cycle, reflecting that *OGG1* expression is not cell-cycle regulated, or induced by oxidative DNA damage. However, we found that oxidative DNA damage accumulation at telomeres promoted the recruitment of BER enzymes to these regions to repair oxidized bases (Fig. 2). These results suggest that, although OGG1 might behave as a housekeeping gene^36,37^, it is actively recruited at telomeres in response to oxidative DNA damage to initiate BER. Our results, together with previous studies, support the role of OGG1 as a key element promoting oxidative DNA damage repair at the telomere, via BER initiation^16,38–40^.

Recently, the first example of a cell-active inhibitor targeting OGG1 has been reported^23^ as well as details on how TH5487 binds to the human OGG1 sensitizing cancer cell lines by inducing replication stress without increasing nuclear 8-oxoG levels^24,25^. In relation with this, the accumulation of 8-oxoG after TH5487 treatment could be expected at guanine enriched regions such as gene promoters, 5ʹ, and 3ʹ untranslated regions, or telomeres. Indeed, here we have found several overlapping phenotypes between OGG1 depletion or inhibition in U2OS cells. First, a disruption in the recruitment of BER elements at telomeres during OS conditions was detected (Fig. 2), resulting in the accumulation of oxidized bases at this region (Supplementary Figure S4B). Although oxidative DNA lesions can potentially be converted into DSBs during BER^41^, we were not able to detect telomere DNA damage response (DDR) because of OGG1 inactivation (Supplementary Figure S5). This result is supported by previous findings showing no DDR induction in OGG1 deficient cells^16^, fewer incisions in TH5487-treated oxidatively stressed cells^24^ or nor a DDR being restricted to cells undergoing replication after TH5487 treatment in leukemic cells^25^. Second, we found that oxidative DNA damage at the telomere in OGG1 inactivated U2OS cells (depleted and/or TH5487-treated), correlated with telomere losses and micronuclei formation (Fig. 3B,D). In line with our results, these phenotypes were also reported in HeLa OGG1-KO cells in which chronic 8-oxoG at telomeres induced telomere replication stress resulting in telomere crisis and the arrest of cell proliferation^16^.

In contrast, we detected minor proliferation defects related to OGG1 deficiency in combination with OS (Fig. 3E). Since some telomerase negative cells show high basal levels of signal-free ends and telomere sister chromatid exchange^42^, which is characteristic of ALT + cancer cells, such as the U2OS cell line^43^, it would be possible that telomere loss is well tolerated in these cells. And although we have recently proposed OGG1 as a potential anti-cancer target, and TH5487 targets a wide range of cancer cells^25^, DepMap Public Database shows that cancer cell lines derived from bone, such as U2OS, present the lowest OGG1 cancer-dependency scores (DEMETER2; p = 6.3 × 10^–6^) among lineages. On the other hand, it has been reported that TH5487 suppresses proinflammatory gene expression^23^. Given that chronic inflammation leads to telomere dysfunction^44^, the telomeric and post-mitotic defects caused by TH5487 could be attenuated by reducing the inflammatory conditions.

Finally, since TH5487 had a low impact on U2OS viability, we combined TH5487 with a heterogeneous set of conventional chemotherapeutic agents and BER inhibitors, looking for potential synergies in telomerase negative (U2OS) or positive transformed human cell lines (BJ-TERT, HCT116, NTUB1). Despite poly ADP ribose polymerase inhibitors (PARPi) targeting BER are known to induce replicative stress at telomeres that leads to telomere crisis^45^, we did not find any synergy or additive effect with TH5487 (Table 1). Indeed, it has been recently described that olaparib toxicity in BRCA1-depleted cells is attenuated by treatments with a ROS scavenger, hypoxia, or OGG1 inhibitor^46^, suggesting that toxicity derived from PARPi in cancer cells might be attenuated by BER inhibition.

Among the different conventional cancer drugs tested, we found methotrexate to present the highest Zero Interaction Potency (ZIP) synergy scores (Z-score > 10 in the 4 tested cell lines). This drug has been reported to induce apoptosis through OS in rat small intestine^47^, psoriasis patients^48^, or lymphocytic T cells^49^. In line with these findings, we showed that methotrexate induced intracellular ROS formation in all the cell lines we tested (Fig. 4). Although methotrexate and TH5487 consistently synergized and correlated with intracellular ROS formation, the molecular mechanisms explaining the effects on cell viability for this drug combination were found to be cell type-specific and very little dependent on telomere instability or micronuclei formation in some cell lines, such as BJ-TERT, HCT116 or NTUB1.

On the other hand, we have reported that pharmacological inhibition of OGG1 with TH5487 might improve the anticancer properties of methotrexate in U2OS cells, partially through an additive or synergistic increase in telomere DNA damage (53BP1 overlapping with TRF2) and genome instability (micronuclei formation) (Fig. 5), which is potentially associated with methotrexate ROS induction. Supporting this, micronuclei formation in U2OS cells was found to increase upon OS especially in OGG1 inhibited/depleted U2OS (Fig. 5C). In contrast, telomere DNA damage in U2OS cells after combining TH5487 and methotrexate (Fig. 5A) might be independent of methotrexate ROS induction, since we did not find additive effects on 53BP1-TIF formation when combining TH5487 with H_2_O_2_ (Supplementary Figure S5). Alternatively, this phenotype could be indirectly mediated by methotrexate folate metabolism^50^ since methotrexate inhibits folic acid synthesis which has been reported to decrease intracellular ROS levels, telomeric DNA oxidative damage, and TIFs in astrocytes^51^.

Combination therapies between TH5487 and methotrexate might be useful for those cancer types in which methotrexate is indicated such as breast cancer, acute lymphatic leukemia, lung cancer, lymphoma, and osteosarcoma^52^. Besides cancer, there are some autoimmune diseases that are treated with methotrexate such as psoriasis, rheumatoid arthritis, and Crohn's disease^52^. Methotrexate in combination with tumor necrosis factor (TNF) inhibitors induces high remission rates in rheumatoid arthritis patients^53^ or higher drug survival in psoriatic arthrosis patients^54^. In this regard, TH5487 is also known to prevent tumor necrosis factor-α-induced OGG1-DNA interactions that mediate TNF related pro-inflammatory gene expression^23^. Hence, considering TH5487 as a “TNF-like inhibitor” we could expect some advantages of combination therapies in autoimmune diseases as well.

To conclude, our results show that TH5487 recapitulates some of the telomeric and post-mitotic defects previously reported in OGG1 KO cells^16^. Therefore, OGG1 inhibitors can be considered as a new tool to block BER and to induce oxidative DNA damage at telomeres inducing cancer cell death, alone or in combination with other drugs like methotrexate. This combination could be especially interesting to overcome methotrexate resistance in cancer or autoimmune diseases^55,56^. In conclusion, our data not only illustrate the importance of BER in oxidative DNA damage repair at telomeres but also point to the possible use of the OGG1 inhibitor TH5487, alone or in combination with methotrexate, to induce telomere instability and proliferation defects, with potential implications in cancer treatment.

## Methods

### Cell culture and treatments

U2OS and BJ-TERT cells were cultured in Dulbecco's Modified Eagle Medium (DMEM; Lonza or Gibco) growth medium while NTUB1 and HCT116 were cultured in RPMI 1640 (Gibco) and McCoy's 5A Medium (Gibco), respectively. All cell lines were supplemented with 10% of fetal bovine serum (Biowest) and 100 U/ml penicillin–streptomycin (Gibco) and grew at 37 °C in a 5% CO_2_ atmosphere. The U2OS human osteosarcoma cell line was obtained from the commercial supplier American Type Culture Collection (ATCC). The HCT116 human colon carcinoma cells were obtained from Dr. Bert Vogelstein (Johns Hopkins, Baltimore, MD). The NTUB1 human bladder carcinoma cells were obtained from Dr. TC Lee (Academia Sinica Taiwandisabled, Nankang, Taiwan). The BJ-Tert cell lines were provided by Dr. W. Hahn (Dana-Farber Cancer Institute).

To induce oxidative DNA damage, cells at about 80% of confluence were treated with H_2_O_2_ (Sigma) at 200 µM in serum-free DMEM for the indicated periods. To perform OGG1 inhibition cells were released into fresh medium containing TH5487^23^ or DMSO (Sigma) for the indicated times and concentrations. After treatment, the cells were allowed to recover in complete growth medium for 1 h when mentioned. The different cell lines used for each experiment are detailed in Supplementary Table S1. Cell line authentication was performed by short tandem repeat (STR) Profiling, and mycoplasma testing was performed regularly.

### Plasmid construction OGG1-GFP and transfection

OGG1-GFP vector was generated according to the protocol described in Visnes et al.^23^. U2OS cells were transfected with the vector using jetPEI (Polyplus) and selected with 1 µg/ml puromycin for 10 days. This was followed by clonal expansion to generate a single clone of U2OS cells constitutively expressing OGG1-GFP, and thus variability in expression levels was minimized.

### CRISPR/Cas9 knockout of OGG1

sgRNAs were designed using the Benchling CRISPR sgRNA Design tool (http://www.benchling.com). A specific sgRNA was tested against *OGG1* gene and also a non-targeting (NT) control was used (sgOGG1#1: GTGTACTAGCGGATCAAGTA and sgNT: CCGCGCCGTTAGGGAACGAG). Those sequences were cloned into the lentiCRISPRv2 vector (Addgene plasmid #52961) and verified by Sanger sequencing.

Viruses were produced by transient plasmid transfection into 293 T cells by the calcium phosphate method, as previously described^57^. Briefly, cells were seeded at 1.1 × 10^7^ cells/dish in 15-cm dishes the day before transfection. U2OS OGG1-GFP cells were transfected using second-generation packaging plasmids (psPAX2 and pMD.2G, Addgene #12260 and #12259, respectively) and the appropriate transfer plasmid (pLV CRISPR sgOGG1 or sgNT). The medium was collected after 48 h, cleared by low-speed centrifugation, and filtered through 0.45 µm-pore-size PVDF (polyvinylidene difluoride) filters (Millipore). Viral titers were calculated and values ranged around 10^7^ to 10^8^ TU/ml. In order to carry out transductions, cells were split and 24 h later were transduced using a multiplicity of infection (MOI) of 5 to ensure a high rate of transduced cells. Cells were incubated at 37 °C for 12 h, and viral supernatant was replaced with fresh cell medium. A sorting step of the GFP-negative cells was carried out to finally obtain the pool of cells where *OGG1* knockout was validated by immunoblotting and IF (further detailed protocol).

### IF microscopy and image analysis

U2OS cells were seeded in a 12-well plate for 24 h before the start of the indicated treatments and followed by the IF protocol. Before fixation, cells were previously extracted with 0.2% Triton X-100 in PBS (phosphate buffered saline; Sigma) for 2 min (pre-extraction). Cells were fixed with 4% paraformaldehyde (PFA; Agar Scientific) for 10 min. After washing with PBS (Sigma), cell permeabilization was performed with 0.5% Triton X-100 (Sigma) in PBS for 15 min. Blocking with 3% bovine serum albumin (BSA; Sigma) in PBS for 1 h was followed by staining with primary and secondary antibodies and 0.5 µg/ml 4′,6-Diamidino-2-phenylindole dihydrochloride (DAPI; Sigma). After each staining, a washing step three times (10 min in PBS each time). Primary antibodies used were mouse anti-TRF2 (ab13579, Abcam) at 1/200, with antirabbit anti-γH2AX (2577S; Cell Signalling), anti-53BP1 (ab36823, Abcam) at 1/1000, anti-XRCC1(ab134056; Abcam) at 1/200. Secondary antibodies: Anti-mouse Alexa 555 (ThermoFisher Scientific), anti-rabbit Alexa 647 (ThermoFisher Scientific). All steps were performed at room temperature. Image acquisition was performed with a Leica confocal microscope SP5 using ACS APO 40.0 × 1.15 OIL lens. Image treatment was done with Leica and ImageJ software and the analysis was performed using CellProfiler software. For the analysis, we evaluated mean signal intensity within telomeres for OGG1-GFP and XRCC1 as previously described^16^ (BER activation at telomere). For DNA damage markers γH2AX and 53BP1, we measured overlapping index with the telomere marker TRF2. Finally, micronuclei frequency was calculated using CellProfiler software. All the experiments were performed at least 2 independent times. Data is available.

### Telomere fluorescence in situ hybridization (Telo-FISH)

Cells were treated with 0.2 µg/ml Colcemide (Life Technologies) for 4 h to enrich cells at metaphase. Cell pellets were exposed to hypotonic treatment with 75 mM KCl solution, fixed in cold Carnoy’s solution [methanol:acetic acid (3:1)], and spread onto glass slides. The samples were fixed again in PBS containing 3.7% PFA and dehydrated by successive incubations in 70, 80, and 100% ethanol before FISH hybridization. DNA was denatured at 72 °C in 1 M HCl, 20 × saline-sodium citrate (SSC), and deionized formamide hybridization mixture, and hybridized with Cy3-labeled (CCCTAA)_3_ peptide nucleic acid (PNA) telomere probe (0.5 μg/ml) [Panagene, PNA BIO/F1001 (TelC-FAM)]. Finally, the slides were washed with a buffer containing the same high percentage of formamide to remove the non-specifically bound probe, and DNA was stained with 0.5 µg/ml DAPI/Antifade solution (Palex Medical). Telo-FISH images were digitally acquired with a CCD camera (Photometrics SenSys) connected to a Leica DM5500B microscope using a 100 × objective and using CytoVision software 7.2. Images were blindly analyzed to score for chromosome multi-telomeric signal or signal-free ends.

### Cell sorting

U2OS cells were trypsinized, resuspended at a concentration of 5 × 10^6^ cells/ml and incubated with 5 µg/ml Hoechst for 15 min at 37 °C in the dark. Cells were sorted based on the amount of DNA by defining three regions for sorting: G1, S, and G2/M phases. A post-sorting purity check was used to confirm the purities of the resulting sorted populations that were higher than 90% in all cases (Supplementary Figure S1A). The sorting was performed with the use of a BD Influx (BD Biosciences). The separated cells (at least 1 × 10^6^ cells from each sorted population) were collected in tubes containing 0.5 ml culture medium and after centrifugation, cell pellets were stored at − 20 °C until used for DNA or protein extraction.

### Colony formation assay

U2OS cells (OGG1-GFP or OGG1-KO) with DMSO or with the indicated concentration of TH5487 were counted and seeded on 10 cm Petri dishes (500 cells per dish) and incubated until colony size surpassed a minimum of 50 cells (6 days). Then, the medium was removed, and cells were challenged with a single pulse of OS H_2_O_2_ (Sigma) at 200 µM in serum-free DMEM for 1 h). Next, treatment was removed, and cells were plated in complete medium in the presence or absence of TH5487 (10 µM) for 6 additional days. Finally, cells were washed twice with PBS, fixed with ice-cold methanol (Sigma) for 5 min, and stained with 1% crystal violet solution (Sigma) for 30 min. Following extensive washes in tap water and air drying. Plates were scanned and relative colony area was measured with ImageJ software. This experiment was performed once. Data is available.

### DNA extraction, human OGG1 purification and relative quantification of oxidized bases in specific genome regions by quantitative PCR (qPCR)

DNA was extracted from cultured cells using the Flexigene DNA Kit (Qiagen) following the manufacturer’s instructions and quantified by the PicoGreen fluorometric assay (Thermo Fisher Scientific).

We have adapted the telomere oxidation protocol previously described^27^ to quantify the relative accumulation of oxidized bases in specific genome regions by incubating the DNA with hOGG1 protein, which was previously purified as previously reported^23^. This is a qPCR method which is based on differences in PCR kinetics between template DNA digested by OGG1 and undigested DNA. This enzyme recognizes and cuts 8-oxoG, producing abasic sites that are converted into SSBs by its AP lyase activity. These SSBs inhibit the PCR, thus, the ΔCt after digesting DNA by OGG1 (Ct digested–Ct undigested) is proportional to the oxidative damage in the amplified region (Supplementary Figure S1B). Conditions used for incubation were 2.4 μM hOGG1 for 4 h in DNA glycosylase buffer (25 mM Tris–HCl, 15 mM NaCl, 2 mM MgCl_2_, 0.0025% Tween-20 at pH = 8). The reaction was stopped by incubating at 95 °C for 5 min. qPCR analysis was performed on 40 ng of digested or undigested genomic DNA using the same reagents, primers, and conditions as described in the original protocol^27^. Each qPCR was performed in triplicate including no-template controls in an Abi QuantStudio 6 Flex Real-Time PCR System (Applied Biosystems). Primers used are listed in Supplementary Table S2. Six independent experiments were included for each condition and analyzed in triplicate. Data is available.

### Protein extraction, quantification, and immunoblotting

Protein expression was determined by immunoblotting. Briefly, cell pellets were prepared in radioimmunoprecipitation assay (RIPA) buffer (Sigma) in the presence of a protease inhibitor cocktail (Roche). Total protein concentration was determined using the Pierce BCA Protein Assay Kit (Thermo Fisher Scientific) following the manufacturer’s instructions. Forty micrograms of protein were electrophoresed on 12% sodium dodecyl sulfate–polyacrylamide gel electrophoresis (SDS-PAGE) and transferred to Immobilon-FL membranes (Millipore). Membranes were blocked in tris buffered saline with Tween-20 (TBS-T; 50 mM Tris/HCl, 150 mM NaCl, pH 7.5 plus 0.2% Tween-20) and 5% non-fat milk for 1 h at room temperature. Blots were probed with the following primary antibodies: rabbit anti-OGG1 (ab124741, Abcam) at 1/2500 dilution, and mouse anti-β-actin (A5441; Sigma) at 1/10,000 dilution in TBS-T containing 5% non-fat milk. Anti-mouse and anti-rabbit IgG-HRP (immunoglobulin G horseradish peroxidase; Dako) were used as the secondary antibodies, and the immunoblots were developed using Immobilon Classico Western HRP substrate (Millipore). Each immunoblot was performed in triplicate. Images were analyzed using ImageJ software (NIH Image), and OGG1 protein level was normalized to actin levels. The full-length blots are presented in Supplementary Figure S2.

### Detection of intracellular ROS during cell cycle phases by flow cytometry

The generation of intracellular ROS during the cell cycle was determined using the fluorescent probe 2′,7′-dichlorodihydrofluorescein diacetate (H2DCFDA; Molecular Probes) combined with Hoechst staining for detecting DNA content. The non-fluorescent H2DCFDA passively diffuses into cells and is converted to the highly fluorescent 2′,7′-dichlorofluorescein (DCF) upon oxidation by ROS. Cells were harvested using Trypsin (1×) for 5 min, pelleted and resuspended in PBS containing Hoechst (1 µg/ml) for 15 min. Then, cells were washed with PBS and pelleted by centrifugation. Next, pellets were resuspended in RPMI without serum-containing H2DCFDA to a final concentration of 10 μM, cells were incubated for 30 min at 37 °C and analyzed by flow cytometry (Navios, Beckman Coulter) using the FL1 (525/540 nm) or FL9 (450/460 nm) channels. We used the median value of H2DCFDA intensity as a threshold to stratify negative (below median) or positive (above median) cells. Then, the percentage of ROS positive cells in G1, S, or G2M phases was calculated. This experiment was performed two independent times. Data is available.

### Chromatin immunoprecipitation (ChIP)

ChIP was performed as previously reported^58^ in parental U2OS cells or U2OS OGG1-GFP cells. Chromatinized OGG1-GFP protein fraction was enriched by using GFP-Trap for immunoprecipitation (Chromotek). DNA bound to OGG1-GFP was heated to reverse crosslinking. The purified OGG1-GFP DNA was amplified by PCR both telomere sequence and the single-copy gene *36B4* using specific primers (Supplementary Table S2). OGG1-GFP enrichment at telomeres or *36*B4 normalized to the 10% input was used to calculate the relative OGG1 enrichment for the 2 regions in U2OS-GFP cells compared to the parental U2OS cells. This experiment was performed 2 times. Data is available.

### OGG1 target engagement

For sample preparation, cells were incubated with 20 µM TH5487 for 2 h at 37 °C, before they were submitted for 3 min at twelve different temperatures ranging from 37 to 62 °C. After the addition of lysis buffer [50 mM Tris–HCl pH 7.5, 150 mM NaCl, 1 mM ethylenediaminetetraacetic acid (EDTA), 1% NP-40, 0.5% sodium deoxycholate and 0.1% SDS supplemented with complete protease inhibitor cocktail (Roche)], cell lysis by freezing–thawing took place. In the following centrifugation (30 min at 17,000*g* at 4 °C) was performed and 70 µL of the supernatant was mixed with 23 µL loading dye before the samples were heated at 95 °C for 10 min. In the following, SDS-PAGE and WB were performed. The membrane was blocked with 5% skimmed milk for 1 h at room temperature. As primary antibodies rabbit anti-OGG1 (ab124741, Abcam) 1:1,000 and mouse anti-Actin (ab6276, Abcam) 1:5,000 were used. This experiment was performed once in U2OS cells (parental) and once in U2OS OGG1-GFP cells (not shown). Data is available.

### Synergy experiments

Drug combinations were built and dispensed using D300e Digital Dispenser (Tecan) in 96 or 384 well plates. Cells were seeded in 96- or 384-well plates containing drug combinations by using Multidrop Combi Reagent Dispenser (ThermoFisher Scientific). Then, cells were incubated for 3 days at 37 °C. Resazurin (R7017, Sigma) was added to a final concentration of 0.01 mg/ml resazurin and fluorescence was measured at ex530/em590 after incubation for 6 h in an Hidex Sense microplate reader (Hidex). Drug synergy Z-score was calculated and interpreted using Synergy Finder (http://synergyfinder.fimm.fi).

A preliminary synergy screening in U2OS, BJ-TERT, NTUB1, and HCT116 cells for TH5487 with conventional chemotherapeutic drugs (cisplatin, 5-Fluoracil, doxorubicin, methotrexate) or BER inhibitors (olaparib, APE1i) was initiated to select the candidates. This initial screening was performed once. For the best candidates, methotrexate and doxorubicin synergies were repeated three independent times in four independent cell lines (U2OS, BJ-TERT, HCT116 and NTUB1).

### Statistical analysis

The Kolmogorov–Smirnov test was used to evaluate whether the data sets were normally distributed. For comparative analyses, statistically significant differences were assessed by an unpaired t-test for normal distributions and the Mann–Whitney U-test for non-normal distributions. Statistical calculations and graphs were done using the SPSS software package version 19.0 (IBM) and GraphPad Prism 8 (GraphPad Software Inc).

## Supplementary Information

Supplementary Information.

## Data Availability

The datasets used and analysed during the current study are available from the corresponding author upon reasonable request.

## Abbreviations

53BP1: P53-binding protein 1
8-oxoG: 8-Oxoguanine
ALT: Alternative lengthening of telomeres
APE1i: Apurinic/apyrimidinic endonuclease 1 inhibitor
BER: Base excision repair
BSA: Bovine serum albumin
ChIP: Chromatin Immunoprecipitation
DAPI: 4′,6-Diamidino-2-phenylindole
DCF: 2′,7′-Dichlorofluorescein
DDR: DNA damage response
DMEN: Dulbecco's modified eagle medium
DMSO: Dimethyl sulfoxide
DSB: Double-strand break
EDTA: Ethylenediaminetetraacetic acid
GFP: Green fluorescent protein
H2AX: H2A histone family member X
H2DCFDA: 2′,7′-Dichlorodihydrofluorescein diacetate
IF: Immunofluorescence
IgG-HRP: Immunoglobulin G horseradish peroxidase
KO: Knockout
LIG3: DNA ligase III
MOI: Multiplicity of infection
NT: Non-targeting
OGG1: 8-OxoG DNA glycosylase 1
OS: Oxidative stress
PARPi: Poly ADP ribose polymerase inhibitor
PBS: Phosphate buffered saline
PFA: Paraformaldehyde
PVDF: Polyvinylidene difluoride
PNA: Peptide nucleic acid
POLB: Polymerase beta
qPCR: Quantitative polymerase chain reaction
RIPA: Radioimmunoprecipitation assay
ROS: Reactive oxygen species
SDS-PAGE: Sodium dodecyl sulfate–polyacrylamide gel electrophoresis
SEM: Standard error of the mean
SSB: Single-strand break
SSC: Saline-sodium citrate
STR: Short tandem repeat
TBS-T: Tris buffered saline with Tween-20
Telo-FISH: Fluorescence in situ hybridization
TIF: Telomere dysfunction-induced foci
TNF: Tumor necrosis factor
TRF2: Telomeric repeat-binding factor 2
XRCC1: X-ray repair cross-complementing 1
ZIP: Zero interaction potency
