# Supplementary material for: Small molecule inhibitor of OGG1 blocks oxidative DNA damage repair at telomeres and potentiates methotrexate anticancer effects
Source: Sci Rep. 2021 Feb 10;11:3490. doi: 10.1038/s41598-021-82917-7 (PMC7876102; doi:10.1038/s41598-021-82917-7)
Supplement: Supplementary file 1 — Supplementary Information. [file 41598_2021_82917_MOESM1_ESM.pdf]

SUPPLEMENTARY DATA

Small molecule inhibitor of OGG1 blocks oxidative DNA damage repair at telomeres and potentiates Methotrexate anticancer effects

Author names: Juan Miguel Baquero[1,†], Carlos Benítez-Buelga[2,†,*], Varshni Rajagopal[2], Zhao Zhenjun[2], Raúl Torres-Ruiz[3,4], Sarah Müller[2], Bishoy M. F. Hanna[2], Olga Loseva[2], Olov Wallner[2], Maurice Michel[2], Sandra Rodríguez-Perales[3], Helge Gad[2,5], Torkild Visnes[6], Thomas Helleday[2,5], Javier Benítez[1,7,8] and Ana Osorio[1,7,*]

Addresses:

[1] Human Genetics Group, Human Cancer Genetics Programme, Spanish National Cancer Research Centre (CNIO), Madrid, 28029, Spain

[2] Science for Life Laboratory, Department of Oncology-Pathology, Karolinska Institutet, Solna, 17121, Sweden

[3] Molecular Cytogenetics Group, Human Cancer Genetics Programme, Spanish National Cancer Research Centre, Madrid (CNIO), 28029, Spain

[4] Josep Carreras Leukemia Research Institute, Department of Biomedicine, School of Medicine, University of Barcelona, Barcelona, 08036, Spain

[5] Weston Park Cancer Centre, Department of Oncology and Metabolism, University of Sheffield, Sheffield S10 2RX, United Kingdom

[6] Department Biotechnology and Nanomedicine, SINTEF Industry, Trondheim, N-7465, Norway

[7] Spanish Network on Rare Diseases (CIBERER), Madrid, 28029, Spain

[8] Human Genotyping-CEGEN Unit, Human Cancer Genetics Programme, Spanish National Cancer Research Centre, Madrid (CNIO), 28029, Spain

*To whom correspondence should be addressed. Tel: +34 91 732 8002; Fax: +34912246980; Email: aosorio@cnio.es. Correspondence may also be addressed to Carlos Benítez-Buelga. Tel: +46700248453; Email: carlos.benitez-buelga@scilifelab.se

[†]The authors wish it to be known that, in their opinion, the first two authors should be regarded as joint First Authors.

SUPPLEMENTARY TABLES

Supplementary Table S1. Cell lines used in principal or supplementary figures

Principal Figures	Cell lines	Supplementary Figures	Cell lines
Figure 1	U2OS parental and OGG1-GFP	Supplementary Figure S1	U2OS
Figure 2	U2OS OGG1-GFP and U2OS OGG1-KO	Supplementary Figure S2	U2OS parental, OGG1-GFP and OGG1-KO
Figure 3	U2OS OGG1-GFP and U2OS OGG1-KO	Supplementary Figure S3	U2OS parental and OGG1-GFP
Figure 4	U2OS, BJ-TERT, NTUB1, HCT116	Supplementary Figure S4	U2OS parental, OGG1-GFP and OGG1-KO
Figure 5	U2OS	Supplementary Figure S5	U2OS OGG1-GFP and U2OS OGG1-KO
		Supplementary Figure S6	U2OS OGG1-GFP and U2OS OGG1-KO
		Supplementary Figure S7	U2OS, BJ-TERT, NTUB1
		Supplementary Figure S8	NTUB1, BJ-TERT

Supplementary Table S2. List of primers used in this paper

**Target**	**Oligo sequence**
36B4-F	5’ CAGCAAGTGGGAAGGTGTAATCC 3’
36B4-R	5’ CCCATTCTATCATCAACGGGTACAA 3’
Telomeres-F	5’ CGGTTTGTTTGGGTTTGGGTTTGGGTTTGGGTTTGGGTT 3’
Telomeres-R	5’ GGCTTGCCTTACCCTTACCCTTACCCTTACCCTTACCCT 3’

SUPPLEMENTARY FIGURES

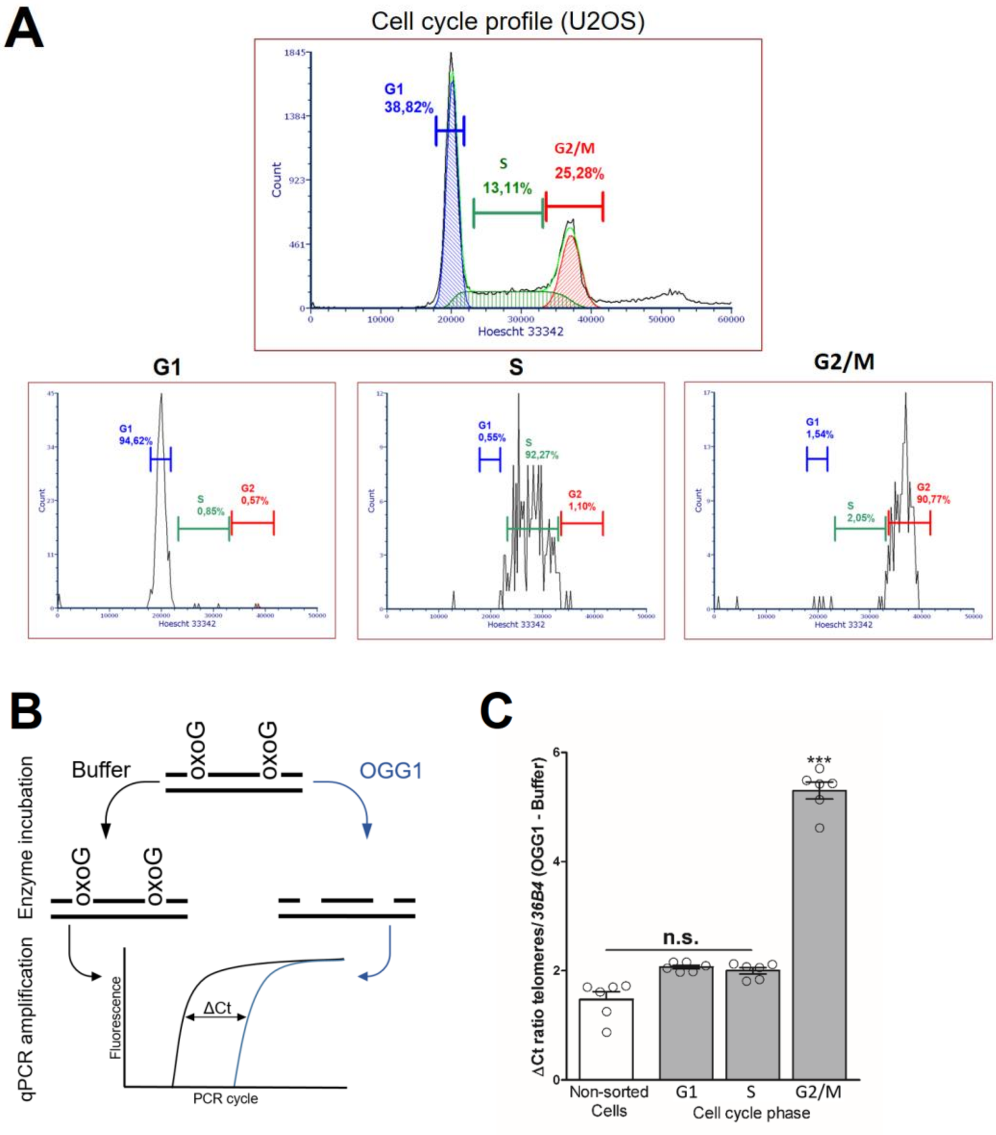

Supplementary Figure S1. (**A**) U2OS cell cycle profile and the establishment of sorted-cells populations according to cell cycle phases (G1, S, G2/M). Post-sort purity check of the resulting sorted populations. The purity was higher than 90% in all cases. (**B**) Schematic representation of the qPCR-based method to evaluate the oxidative DNA damage within specific DNA amplified regions. This methodology is based on differences in PCR kinetics between DNA template digested by DNA glycosylases OGG1 and undigested DNA. (**C**) Relative levels of telomeric oxidized bases between telomeres and the *36B4* locus (ΔCt telomeres/ΔCt *36B4*) throughout the cell cycle phases (G1, S, and G2/M). Bars show the mean and the standard error of the mean (SEM) from 3 technical replicates from 6 independent experiments for each condition (two-sided T-test; *** P<0.001).

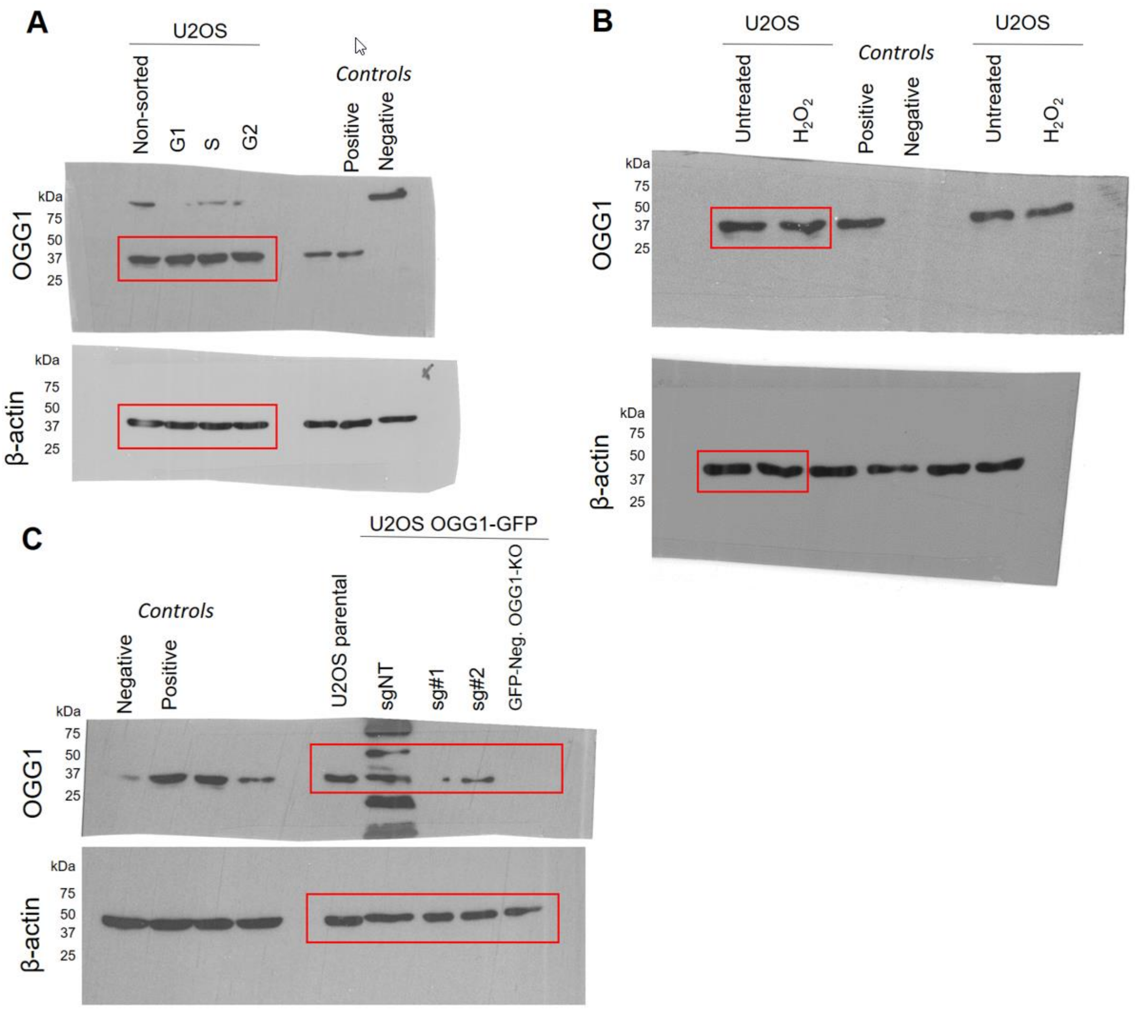

Supplementary Figure S2. Full-length Western blot images. (**A**) Western blot from Figure 1D. (**B**) Western blot from Supplementary Figure S3B. (**C**) Western blot from Supplementary Figure S4D. The red boxes denote the regions used in the corresponding main and supplementary figures.

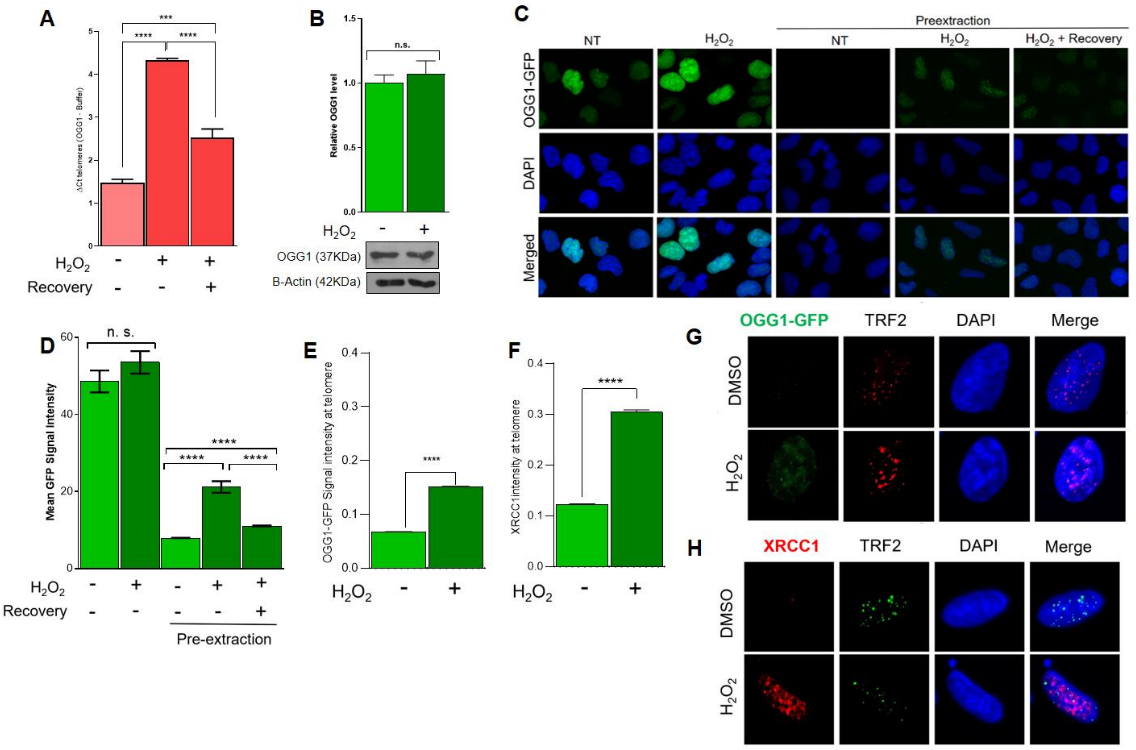

Supplementary Figure S3. (**A**) DNA from U2OS cells was used to evaluate 8-oxoG levels at telomeric DNA after OS treatment (H_2O_2 200µM/1h) and, after OS treatment followed by a recovery period (fresh medium/1h). Bars show the mean and the SEM from 3 technical replicates from 6 independent experiments for each condition (two-sided T-test; *** P<0.001). (**B**) Quantification of OGG1 protein expression level in U2OS cells in response to OS treatment (H_2O_2 200µM/1h). Actin levels were used to normalize for protein loading. Immunoblot was performed in duplicate (two-sided T-test). The full-length blots are presented in Supplementary Figure S2. (**C**) Confocal microscopy images showing OGG1-GFP staining pattern (green) within the nucleus, stained in blue with DAPI. After pre-extraction, soluble proteins are removed and no OGG1-GFP signal can be detected without oxidative treatment (H_2O_2 200µM/1h). (**D**) Quantification of OGG1-GFP signal intensity for the conditions presented in (C). (**E**) Comparative analysis and bar graph for average ±SEM of OGG1-GFP signal intensity contained within TRF2 foci. (**F**) Comparative analysis and bar graph for average ±SEM of XRCC1 signal intensity contained within TRF2 foci. Signal intensity comparative analysis in (D), (E), and (F) included at least 200 cells/exp in 2 independent experiments. (two-sided T-test; ***P<0.001 and ****P<0.0001). (**G**) Confocal images showing OGG1-GFP staining pattern (green) and telomere TRF2 (red) within the nucleus, stained in blue with DAPI. (**H**) Confocal images showing XRCC1 staining pattern (red) and telomere TRF2 (green) within the nucleus, stained in blue with DAPI.

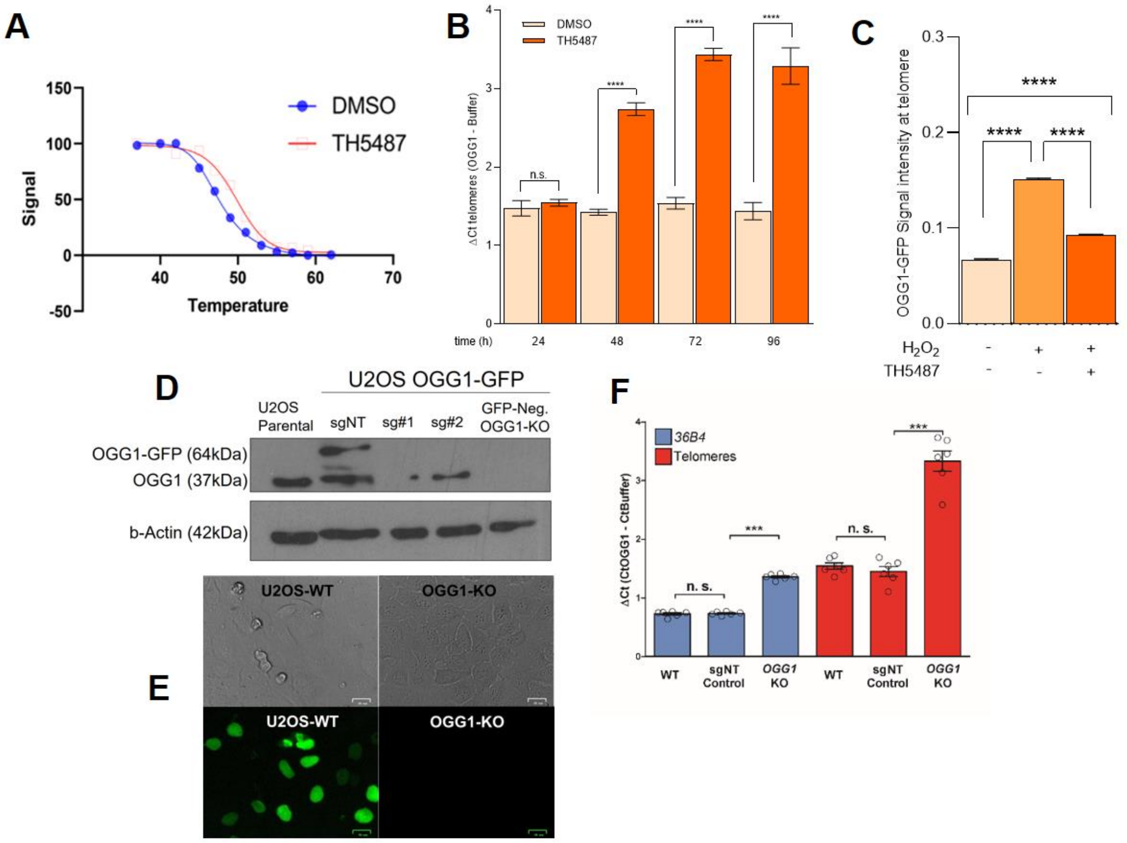

Supplementary Figure S4. (**A**) Comparative graph showing a shift in the Thermal stabilization profile for U2OS cells treated with DMSO or TH5487. (**B**) DNA from U2OS cells was used to evaluate 8-oxoG levels at telomeric DNA after TH5487 treatment (10µM) during the indicated periods (hours). Bars show the mean and the SEM from 3 technical replicates from 4 independent experiments for each condition (two-sided T-test; *** P<0.001). (**C**) OGG1-GFP intensity at telomere (TRF2) after oxidative stress treatment (200µM/1h H_2O_2) alone or in the presence of 10 µM TH5487. Data are the average with SEM from 2 independent experiments (two-sided T-test; ****P<0.0001). (**D**) CRISPR/Cas9 *OGG1* knockout validation by Western blot in U2OS OGG-GFP cells. A sorting step of the GFP negative cells from OGG1 sg1 was carried out to obtain the pool of GFP negative cells validated as OGG1-KO. β-actin was included as loading control. The full-length blots are presented in Supplementary Figure S2. (**E**) Bright-field and fluorescence microscope images showing OGG1-GFP depletion in OGG1-KO cells. (**F**) Relative accumulation of oxidative DNA damage at the *36B4* locus or telomeric DNA in U2OS parental (WT), U2OS OGG1-GFP, and U2OS OGG1-KO cells. Bars show the mean and the SEM from 3 technical replicates from 6 independent experiments for each condition (two-sided T-test; *** P<0.001).

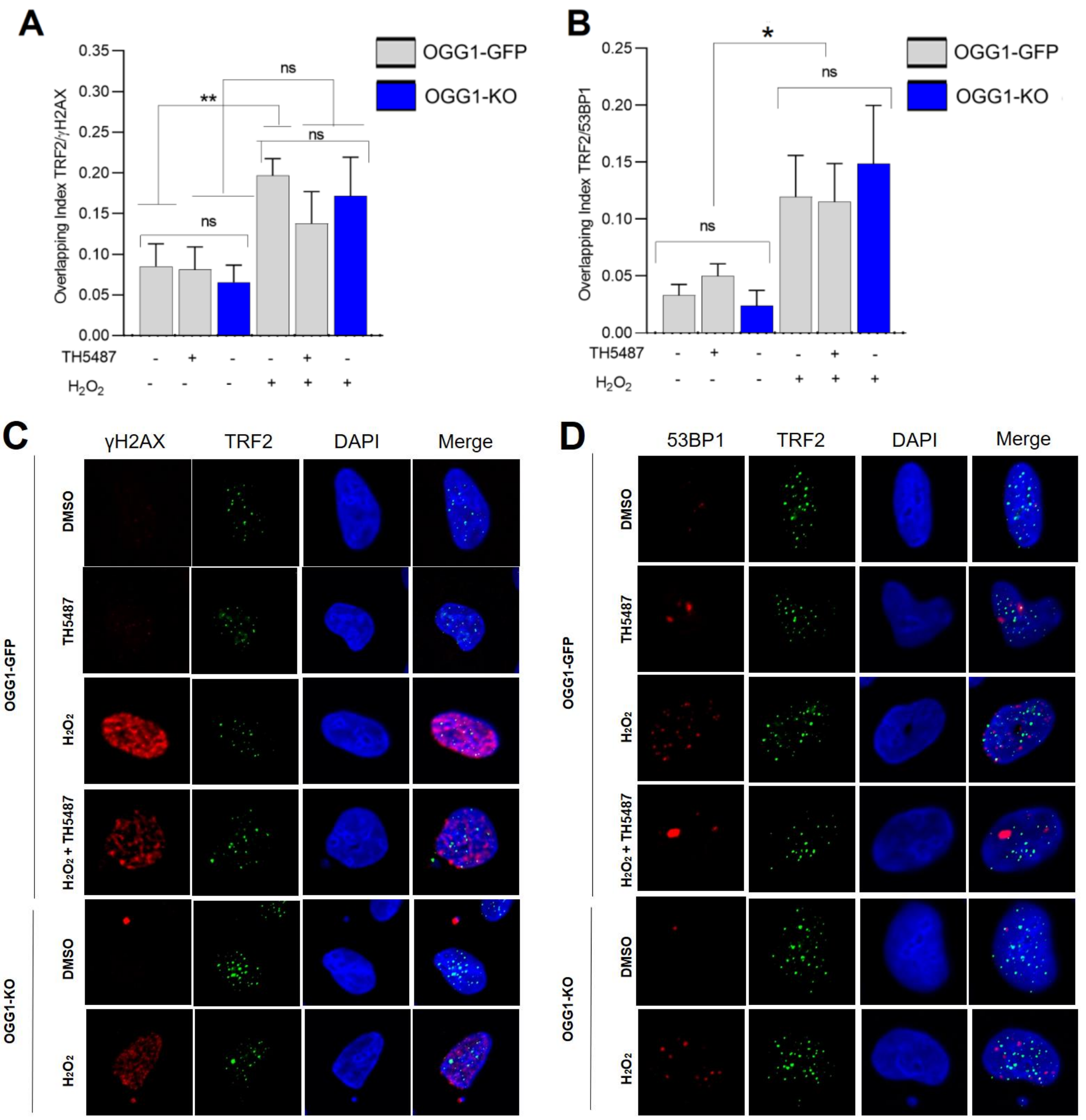

Supplementary Figure S5. (**A**) Quantification of γH2AX signal intensity integrated within telomeres from more than 200 cells per condition. Data are the average with SEM from 2 independent experiments. Significant differences were calculated using the Mann-Whitney test for non-parametric distributions (****P<0.0001). (**B**) Quantification of 53BP1 signal intensity integrated within telomeres from more than 200 cells per condition. Data are the average with SEM from 2 independent experiments. Significant differences were calculated using the Mann-Whitney test for non-parametric distributions (**** p<0.0001). (**C**) Confocal imaging at single cells representative for each treatment condition and stained for γH2AX (red) and TRF2 (green) using specific antibodies or DAPI to stain cell nucleus (blue). (**D**) Confocal imaging at single cells representative for each treatment condition and stained for 53BP1 (red) and TRF2 (green) using specific antibodies or DAPI to stain cell nucleus (blue).

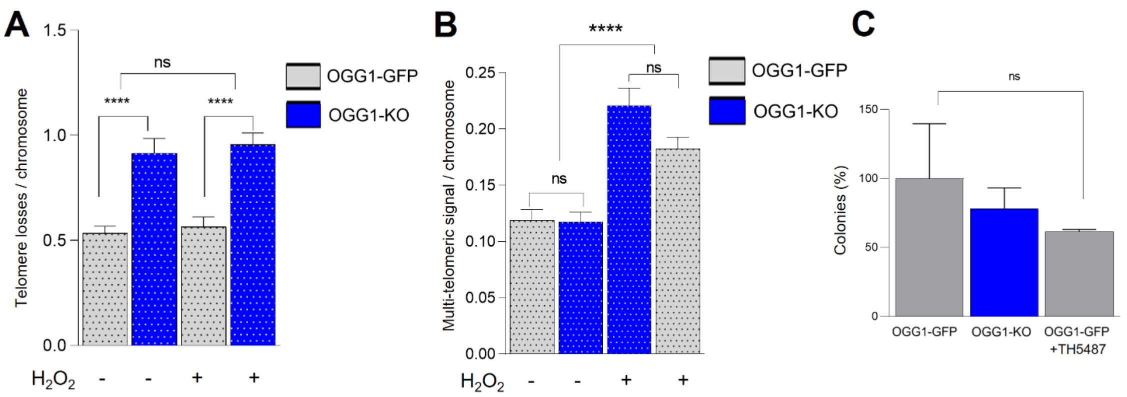

Supplementary Figure S6. (**A**) Quantification of telomeric signal-free ends of metaphase chromosomes for U2OS-GFP or OGG1-KO upon oxidative stress treatment (200µM H_2O_2/1h). Bars show the mean and the standard error of the mean (SEM) for frequency events/metaphase (30 to 35 metaphases per condition from 2 independent experiments). Statistical significance was determined using unpaired, two-sided T-tests (**** $P<0.0001$). (**B**) Comparative analysis of the frequency of multi-telomeric signals for U2OS-GFP or OGG1-KO upon oxidative stress treatment (200µM H_2O_2/1h). Bars show the mean and the standard error of the mean (SEM) for the frequency of events (telomere loss or fragility/Chromosome) in at least 30 independent metaphases per condition from 2 independent experiments. Statistical significance was determined using unpaired, two-sided T-tests (**** $P<0.0001$). (**C**) Comparative analysis for the colony area (pixels) generated in each condition. Bars show the mean and the standard error of the mean (SEM) from a minimum of 45 colonies per condition. Significant differences were calculated using the Mann-Whitney test for non-parametric distributions. This experiment was performed once.

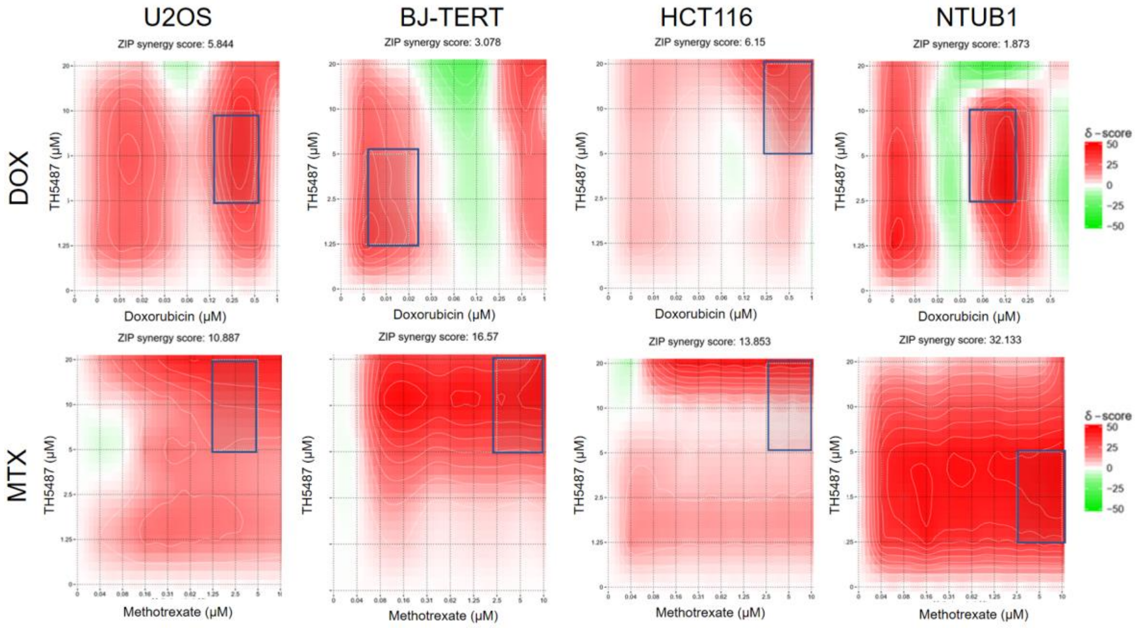

Supplementary Figure S7. Synergy maps showing the drug concentration within the range of maximum synergy (blue rectangle). Maps were generated with the average data from 3 independent experiments. Red to green colour within the map represent ZIP-score values.

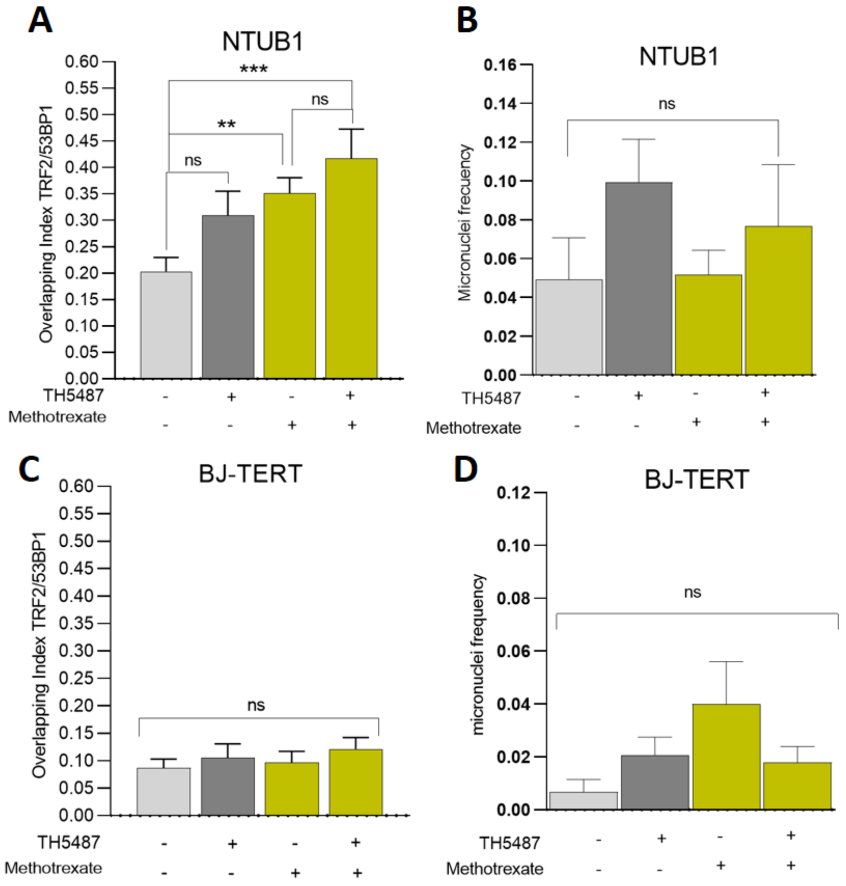

Supplementary Figure S8. (**A**) Quantification of 53BP1 signal intensity integrated within telomeres in NTUB1 or (**C**) BJ-TERT. More than 200 cells per condition. Data are the average with SEM from 2 independent experiments. Significant differences were calculated using the Mann-Whitney test for non-parametric distributions (****P<0.0001). (**B**) Comparative analysis of micronuclei formation frequency for cells incubated with DOX (0.1 µM) or MTX (10 µM) for 72h, alone, or in combination with TH5487 (10µM) in NTUB1 cells or (**D**) BJ-TERT. More than 200 cells per condition were analyzed. Data is the average of 2 independent experiments. Significant differences were calculated using the Mann-Whitney test for non-parametric distributions (**** P<0.0001).
